# Letter from Geo. C. Blackman

**Published:** 1856-10

**Authors:** Geo. C. Blackman

**Affiliations:** Burnet House


*Letter from Geo. C. Blackman.*

BURNET HOUSE, AUG. 4, 1856.

MR. EDITOR:—Since the appearance of the last number of the Lancet, you have assisted me in two operations which for several reasons are worthy of record. I believe with Wiseman, the father of British surgery, that it is the duty of every surgeon to publish his unsuccessful as well as his successful cases. "For my part," he says in his preface, "I have done it faithfully, and thought it no disgrace to let the world see where I failed of success," which failures, he adds, "have attended all my brethren as well as myself, and will attend thee, also reader, in spite of all thy care and diligence, if thou undertake the employment." The date of this preface was May, 1676 (*vide Chirurgical Treatises,*) and after briefly detailing the cases which have occurred in my practice, I propose to determine to what extent the predictions of the "Parê of England" have been verified, as regards the treatment of *old* dislocations of the shoulder.

About the 10th ult., aided by yourself, I succeeded in reducing by manipulation, without the pulleys, a dislocation into the axilla of 80 days' standing. The reduction was accomplished in a very few minutes, under the influence of chloroform and ether, and the next morning the patient left for the country, in a comfortable condition. Since that I have received no tidings from him. Encouraged by the result in this case, another patient, himself a physician, a tall athletic man, and about 50 years of age, decided to submit to the same manipulation, although his arm had been dislocated for about sixteen weeks. The dislocation was downward and inward, and about the 10th week an unsuccessful attempt, by another surgeon had been made with the pulleys, to which the force of six men was applied for two and a half hours. The patient being under the influence of chloroform and ether, aided by yourself, Drs. Fries, Cary, Graham and Kaufman, I commenced my manipulations, adducing, rotating, abducting, and elevating the arm. These efforts had been made for about ten minutes, and the least possible violence employed, when a tumefaction appeared in the pectoral region, which in a few minutes attained considerable size. Supposing that the axillary artery was ruptured, as no pulse could be felt at the wrist, a ligature was immediately applied to the vessel at the upper part of its course. The operation was performed about 10 o'clock, A. M., and compression of the pectoral region, made by means of a sponge and broad roller. On removing this the next morning, the tumefaction had nearly disappeared. The patient continued comfortable, and about nine days after the application of the ligature, I was compelled to leave the city on a professional visit to Indiana. I left on Friday afternoon and returned on Monday morning, at which time I learned that my patient had died on Sunday morning from hemorrhage at the seat of ligature. Two physicians, his most intimate friends, lodged in the same house with him, but before they reached his bedside the quantity of blood lost was so great that he sank exhausted in about two hours from the first and *only* attack of hemorrhage. Previous to my departure, for Indiana, I had suggested to the physicians in charge, the importance of having compressed sponge at hand, to be used in any emergency of the kind, but this was not used by the attendant; instead of applying pressure instantaneously, he went in search of the physicians, who at that early hour in the morning, were in bed. The time thus lost, unquestionably led to the fatal catastrophe.

I might refer you to numerous instances of success in the reduction of old dislocations—from two to six months standing—which have occurred since the days of Wiseman, but I propose to notice only the accidents by which some of these attempts have occasionally been followed. One of the earliest recorded, so far as we have been able to learn, is the case reported by Desault in the *Journal de Chirury.*, t. 4, p. 301.

During the efforts of this surgeon to reduce an old dislocation, suddenly a considerable "*tumeur aerienne*" appeared below the clavicle, which Desault attributed to the "*degagemen de l'air amasse entre les cellules rompues du tissu cellulaire!*" In a few days this tumor entirely subsided under the influence of "*astringens et une compression methodique.*" Whether it was the result of a disengagement of air from the lacerated cells of the cellular membrane, as supposed by Desault, or of a rupture of blood vessels, we leave the reader to determine.

It is somewhat singular that Desault should have met with two cases of this extraordinary phenomenon. Pelletan's explanation, in our opinion, throws some light on this subject. In an attempt to reduce a luxation of four months standing, the same kind of "*tumeur aerienne*" appeared. It was opened, and the hemorrhage from the torn artery was fatal (*Clin. Chirurg.*, t. ii., p. 95.)

Malgaigne states that he is acquainted but with a single instance of an "emphyseme veritable" following a reduction, and that is the one reported by Flaubert, in his *Men. sur. plus cas de luxations dans lesquels les efforts pour la reduction ont ete suivis d'accidents graves;* which appeared in the *Repertoire d'anat. et de phys.*, 1827. The patient, a female, æt. 70, screamed violently during the operation, and Malagigne is disposed to believe that the emphysema was independent of the luxation, or the reduction, (*Traite des Tract. et des Luxations*, tom. ii., p. 147.)

Malgaigne, himself, attempted reduction in a case of sixty-eight days' standing, but was forced to discontinue his efforts in consequence of the sudden appearance of a tumefaction in the axilla, and on the shoulder. Ice was applied, and in the course of a few hours the swelling was arrested, and by the twenty-second day, the blood, which he thinks came from ruptured muscular branches, was completely absorbed, (*op. cit.* page 150.)

A case occurred to Flaubert, in which, besides the tumefaction, the pulse could not be felt at the wrist. The hand was cold, insensible and immovable. The next day, however, the pulse returned to the wrist, and in the course of twenty-six days the effused blood was absorbed. Froriep lost a patient from a rupture of the axillary vein, which proved fatal in an hour and a half after the operation, (op. cit. p. 151.) The reader may find in the comprehensive treatise of Malgaigne, details of cases in which the axillary artery was ruptured. We pass over those observed by Verduc, Petit, Platmer, Delpech, and that referred to by Sir Charles Bell, in his Operative Surgery. The late Dr. John C. Warren tied the subclavian to arrest the progress of an enormous aneurismal tumor in the axilla, the result of the reduction of a recent dislocation, and of supposed pressure of the operator's boot. In this instance the coats of the artery were so contused that sloughing took place during a fit of coughing, five days after the accident—(*Amer. Jour. Med. Sciences*, vol. xi, N. S., 1846.) In 1824, M. Loudet lost a patient at the hospital in Rouen. The dislocation was of only eleven days standing, and was complicated with a fracture of the margin of the glenoid cavity, as in the two fatal cases which occurred in the practice of Prof. Gibson of Philadelphia. The latter cases are too familiar to every surgical student to require particular mention in this place. Prof. Gibson, in connection with the report of the above cases, gives briefly the details of a fatal operation by David, of Rouen. The luxation had existed several months, and great force was employed in the reduction. This resulted in an inflammation, mortification and death. Some years since, Lisfranc attempted the reduction in a case of four months standing. He succeeded; but on visiting the patient an hour afterwards he was found dead. His death was attributed to cerebral congestion, as the autopsy showed the axillary artery, veins and nerves uninjured—Vid. *Bul. de la Soc. de Chirurg. de Paris*, Tom. Prem., p. 718.) In the same volume MM. Lenoir, and Larrey, refer to cases in which they had met with lesion of the brachial plexus, giving rise to paralysis, and yet these were recent cases, and the reduction was most readily accomplished. But I will not multiply cases of this kind; those already related will suffice, in the minds of many, to answer the question—At what period of time after a dislocation of the shoulder, is an attempt at reduction justifiable? When Prof. Gibson lost his first patient, he wrote that "should a case, similar in external appearance to that of James Scofield again occur, I shall feel justified in adopting a similar course."—(*Gibson's Surgery*, vol. 1, p. 824, 5th ed.) When he had lost his second patient (John Langton,) he expressed his views as follows, "The conclusions which I am now prepared to draw, are directly the reverse of what I have stated in some of the foregoing pages; I am now disposed to condemn, in the most unqualified terms, all attempts at the restoration of *ancient* luxations of the humerus and other bones—except in cases where the patient is remarkably thin and debilitated, and where there has been little or no inflmamation at the time or subsequent to the displacement."—(*Op. Cit.* p. 351.) At a meeting of the *Societe de Chirurgie* of Paris, July 3, 1850, M. Maisonneuve reported a case in which, after M. Velpeau had failed, he succeeded in reducing a luxation of the shoulder of 12 weeks standing, and elated with this triumph over the veteran of La Charite, he asserts there are but few cases in which, with the aid of chloroform, we may not succeed. "*Quelles resistances y a-t-il a vaincre ici, en effet?*" He asks, "*Il n'y a presque pas d'engrenage; les muscles sont neutralises par le chloroforme; il ne reste donc que des adherences fibreuses: l'on pourra presque toujours les surnonter les rompre.*"—(*Bul de Soc. de Chir.* t. i. p. 716.) But these fibrous adhesions are not the only obstacles to overcome; where the tissues surrounding the head of the bone have become consolidated by inflammation, the axillary vessels and nerves must be in danger of laceration. Perhaps, however, as M. Maisonneuve suggests, this accident may be avoided by "*extensions preparatoires,*" as in the attempts to restore contracted limbs to their natural shape.

GEO. C. BLACKMAN.

*Western Lancet*

